# Cushing’s syndrome and early growth hormone hypersecretion in a child with Carney complex: a case report

**DOI:** 10.3389/fendo.2026.1843310

**Published:** 2026-06-29

**Authors:** Gaia Pietropaolo, Adalgisa Festa, Giulio Rivetti, Federica Messa, Giovanni Di Iorio, Antonella Klain, Valeria Pellino, Daniela Cioffi, Rosario Ferrigno, Maria Cristina Savanelli, Emanuele Miraglia Del Giudice, Anna Grandone

**Affiliations:** 1 University of Campania “Luigi Vanvitelli”, Department of Woman, Child and of General and Specialized Surgery, Naples, Italy; 2 AORN “Santobono-Pausilipon”, Pediatric Urology Unit, Naples, Italy; 3 AORN “Santobono-Pausilipon”, Endocrinology and Growth Disorders Unit, Naples, Italy

**Keywords:** Carney complex, case report, Cushing’s syndrome, growth hormone hypersecretion, primary pigmented nodular adrenocortical disease

## Abstract

**Introduction:**

Carney complex (CNC) is a rare autosomal dominant syndrome characterized by multiple endocrine and non-endocrine tumors. In childhood, Cushing’s syndrome due to primary pigmented nodular adrenocortical disease (PPNAD) may occur, while growth hormone (GH) hypersecretion before puberty is exceptionally rare.

**Case presentation:**

A 5-year-old girl presented with rapid weight gain, facial changes, hypertension, hypokalemic alkalosis, and kidney stones. Biochemical evaluation confirmed ACTH-independent Cushing’s syndrome, and abdominal magnetic resonance imaging (MRI) revealed bilateral adrenal nodules consistent with PPNAD. Family history of endocrine tumors and cardiac myxomas suggested CNC, subsequently confirmed by genetic testing showing a mutation of the PRKAR1A gene in both the patient and her father. Bilateral adrenalectomy resolved hypercortisolism. At 8.6 years, the patient showed an accelerated growth velocity (+2.48 SDS) with elevated IGF-1 levels and lack of GH suppression during an oral glucose tolerance testing, despite a normal pituitary MRI. She remained asymptomatic apart from growth acceleration, which was carefully monitored during follow-up. Over 18 months accelerated growth persisted with pubertal progression, but IGF-1 levels eventually normalized and brain MRI remained stable; therefore, treatment for GH excess was deferred.

**Conclusions:**

This case highlights the importance of considering CNC in pediatric ACTH-independent Cushing’s syndrome and underlines the role of genetic testing. It also demonstrates that GH hypersecretion may emerge earlier than current screening recommendations, underscoring the need for surveillance starting at the onset of puberty.

## 1 Introduction

Carney complex (CNC) is a rare autosomal dominant multiple endocrine neoplasia and lentiginosis syndrome, characterized by abnormal cutaneous and mucosal pigmentation, myxomas of the heart, skin and breast, schwannomas, and endocrine tumors such as pituitary adenomas, tumors of the adrenal gland, testes, ovaries and thyroid (1, 2).

CNC is principally caused by inactivating mutations or large deletions of the PRKAR1A gene located on chromosome 17q22–24 coding for the regulatory subunit type I alpha of protein kinase A (PKA). Approximately 70% of patients diagnosed with CNC have an affected parent, while 30% have a *de novo* pathogenic variant (2, 3). The median age at diagnosis of CNC is 20 years, but some features, such as abnormal skin pigmentation, can appear since birth (1, 2). During childhood CNC can manifest with cutaneous marks such as lentigines, and some endocrine features including adrenocorticotropic hormone (ACTH) independent Cushing syndrome (CS) due to primary pigmented nodular adrenocortical disease (PPNAD) and large-cell calcifying Sertoli cell tumors (LCCSCTs), which usually manifest during the first or second decade of life. Thyroid nodules and cardiac myxomas can also be detected early (1, 2, 4). In infancy, cardiac and cutaneous myxomas and PPNAD are reported as the most frequent tumors (4). Growth hormone hypersecretion (GHH) usually presents in adulthood with acromegaly, typically in the third or fourth decade of life, whereas it remains a rare manifestation in children (1). GHH in CNC shows considerable clinical heterogeneity and its management depends on clinical severity and MRI findings (5). In this paper we report the case of a 5 years old girl diagnosed with CNC who initially presented with signs and symptoms consistent with CS and subsequently developed early growth hormone (GH) hypersecretion.

## 2 Case presentation

We herein report a rare presentation of CNC in a 5 years old female patient who presented with a 3-months history of rapid weight gain associated with changing in facial physiognomy, asthenia and growth velocity (GV) decrease. The patient was admitted to our department with suspected CS after high levels of free urinary cortisol (>800µg/24h) were detected at external facilities. No history of exogenous steroid use was reported. Physical examination revealed a cushingoid face, acne, hypertrichosis and truncal obesity. The patient weighed 24 kg (+1.45 SDS), height was 105 cm (-1.61 SDS) and BMI was 21 kg/m^2^ (+3.02) with a mid-parental height of 155 cm (**Table 1**). Blood pressure at admission was elevated (+ 2.38 SDS for age and sex) (**Table 1**); this finding was confirmed by 24-hour blood pressure monitoring, which indicated stage 2 hypertension (6). Initial laboratory evaluation included complete blood count, glycemic profile, an evaluation of thyroid and adrenal hormones, urinalysis and an electrolyte panel. The initial biochemical evaluation showed elevated morning cortisol (h 8.00 a.m.) with low levels of ACTH and renin. Aldosterone, thyroid hormones, fasting plasma glucose, blood count and the other adrenal hormones resulted within the normal ranges (**Table 1**). Additional findings included the presence hypokalemic alkalosis (pH 7.47, K^+^ 1.9) and kidney stones which were treated during the hospitalization. Kidney stones were asymptomatic and accidentally found during the abdominal ultrasound (US) performed to study the adrenal glands and localized in the lower calyces of the right kidney with the main stone measuring 12 mm, without evidence of renal dilatation and associated with hypercalciuria. .

**Table 1:** Height SDS refes to Cacciari 2006 growth charts for Italian girls.

Clinical features at CS diagnosis		
Age	5.5	
Height (cm)	105 (–1.61 SDS)	
Weight (kg)	24 (+1.45)	
BMI (kg/m^2^)	21 (+3.02)	
Tanner stage – Breast (B)	1	
Tanner stage – Pubic hair (PH)	1	
Systolic blood pressure (mmHg)	128 (+2.33 SDS)	
Diastolic blood pressure (mmHg)	75 (+1.92 SDS)	
Laboratory findings at CS diagnosis		Reference ranges
Cortisol (μg/dl) h 08.00	55.4	a.m. 4,5-24 p.m. 1,8-6,5
Urinary free cortisol (µg/24h)	>800	< 55
ACTH (pg/ml) h 08.00	5,6	<60
IGF-1 (ng/mL)	134.3	98-396
TSH (µUI/ml)	1.114	0.20-6.1
FT4 (pg/mL)	9.5	6.9-16.9
PRL (ng/ml)	13.5	<25
DHEA-S (µg/mL)	0.2	0.02-0.79
Aldosterone (µg/mL)	3.1	1.76-23.2
Renin (ng/mL/h)	0.2	0.32-1.84
Delta4 Androstenedione (ng/mL)	1.5	<3.164
17-OH Progesterone (ng/mL)	2.2	≤2
Testosterone (ng/mL)	6	<24
Glucose (mg/dl)	83	60-100
Total cholesterol (mg/dl)	176	60-200
LDL (mg/dl)	95	10-129
pH	7.47	7.35-7.45
Sodium (mEq/L)	142	135-146
Potassium (mEq/L)	1.9	3.5-5.3
Calcium (mg/dl)	8.7	8.6-10.2
Creatinin (mg/dl)	0.42	0.3-0.7
Urinary Calcium (mg/24h)	58	100-250

BMI and weight SDS refer to WHO 2006 growth charts for girls >5 years. Blood pressure SDS refer to (6). UFC cut-off is corrected for body surface area.

### 2.1 Diagnosis

To confirm the clinical and biochemical suspicion of CS the patient underwent a low dose dexamethasone suppression test consisting of administration of 1 mg of dexamethasone at night (11:00 p.m.), followed by measurement of cortisol levels the following day at 8.00 a.m. The test showed lack of cortisol suppression consistent with CS, while suppressed basal ACTH levels in morning spot suggested an ACTH independent etiology (7) (**Table 2**). Since differential diagnoses of ACTH-independent CS included adrenocortical tumors and primary adrenocortical hyperplasia, adrenal imaging studies were performed to confirm the source of cortisol production. Abdominal US exam did not reveal adrenal masses, while abdominal magnetic resonance imaging (MRI) confirmed the presence of hypertrophy of both adrenal glands with bilateral evidence of millimetric nodulations consistent with the diagnosis of PPNAD (**Figure 1**). Given the well-established association between primary adrenal hyperplasia and genetic syndromes, a detailed family history assessment was performed. The paternal history of atrial myxoma, thyroid and testicular tumors, cutaneous fibromas, and lentiginous lesions, together with the patient’s presentation with ACTH-independent CS, strongly supported the clinical suspicion of CNC.

**Table 2:** Cortisol values after low-dose Dexamethasone test at diagnosis.

Serum cortisol at midnight and after low-dose Dexamethasone test		Normal values
Serum Cortisol (μg/dl) 08:00 a.m.	51,5	H 08.00 a.m after LDDT <1.8 H oo.oo < 4.4
Serum Cortisol (μg/dl) 00:00 a.m.	45	

**Figure 1:**
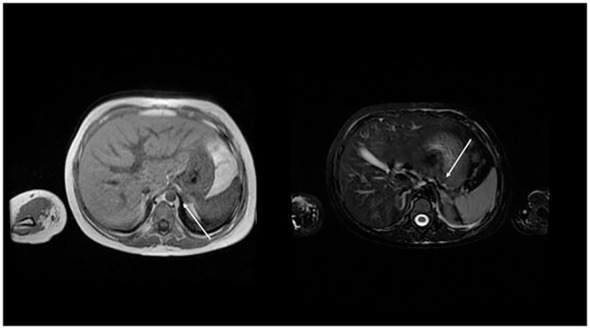
Abdominal MRI showing hypertrophy of both adrenal glands with bilateral millimetric nodulations(arrows).

Sanger sequencing subsequently identified an heterozygous nonsense mutation in exon 3 of the PRKAR1A gene (c.289C>T; p.Arg97Ter). The variant was detected both in the father and the daughter and was compatible with CNC as previously described in literature (8). Given the association between CNC and the development of cardiac myxomas since childhood, a cardiological evaluation with echocardiography was performed, showing no evidence of cardiac involvement.

### 2.2 Treatment and follow-up

Hypertension was treated with ramipril 2,5 mg/die during hospitalization and hypokalemia was corrected with IV therapy. The patient underwent a bilateral laparoscopic adrenalectomy and, after the beginning of a replacement therapy with hydrocortisone and fludrocortisone, a complete regression of the symptoms was obtained. From that point onward, the patient underwent routine instrumental and biochemical follow-up at our institution. At the age of 8.6 years, during a routine follow-up visit the patient exhibited an accelerated GV of 7.35 cm/year calculated over 8 months (+2.48SDS) (**Figures 2**, **3**), prompting further diagnostic investigations to identify the underlying cause (**Table 3**). A complete screening of the pituitary function was performed revealing increased insulin-like growth factor 1 (IGF-1) levels at 464.8 ng/mL (+2.42 SDS), while the remaining pituitary hormones levels were within normal range (**Table 3**). The patient did not report any symptoms or clinical signs suggestive of GHH, except for the accelerated GV In the differential diagnosis of growth acceleration, no additional clinical features were observed, including the absence of pubertal development at that time. Considering that GHH is a recognized manifestation of CNC, albeit typically occurring at a later age, further diagnostic investigations were pursued. To confirm the laboratory finding, an oral glucose tolerance test (OGTT) was performed to assess GH secretion, which demonstrated a lack of GH suppression with a nadir of 1.07 ng/mL (9). Subsequently, the patient underwent MRI of the brain to evaluate the pituitary region. The scan revealed a sella turcica of normal size, with the pituitary gland in its usual location, showing normal morphology, dimensions, and signal characteristics. Bone age was consistent with chronological age. Given the lack of clear recommendations in the literature regarding the management of children with CNC and GHH without pituitary anomalies, and considering the absence of a detectable neoplasia that could be exacerbated by GHH, along with corresponding bone age, normal metabolic parameters and normal cardiovascular screening, we opted for close clinical and biochemical monitoring rather than immediate medical therapy. Treatment options, including somatostatin analogues versus close monitoring, were thoroughly discussed with the parents. Given that accelerated growth velocity was the only manifestation of GHH, the family agreed with a conservative approach. During follow-up, standard age-specific assessments recommended for CNC were performed (2) (**Table 3**). In addition, targeted monitoring for GHH was carried out, including evaluation of auxological parameters, glycemic profile, IGF-1 levels, blood pressure, echocardiography and lipid profile. After 8 months of follow-up, the patient maintained a GV of +2.86 SDS and an IGF-1 of 498.2 ng/ml (+2.25 SDS). In this occasion the patient presented with the first signs of pubertal development (**Table 3**) which might have a role in the acceleration of GV. After 12 months of follow-up, the patient still exhibited progression of pubertal development and accelerated GV (+3.04 SDS), while levels of IGF-1 appeared to be within normal ranges for the first time since diagnosis (399.3 ng/ml, +1.61 SDS). At 15 months follow up IGF-1 was once more above the normal ranges (529.3 ng/ml; +2.44 SDS) with GV of +5.65 SDS. After 18 months, we decided to perform a new brain MRI which showed findings comparable to the previous one, including a pituitary gland in its normal location with regular morphology, size, and signal.

**Figure 2:**
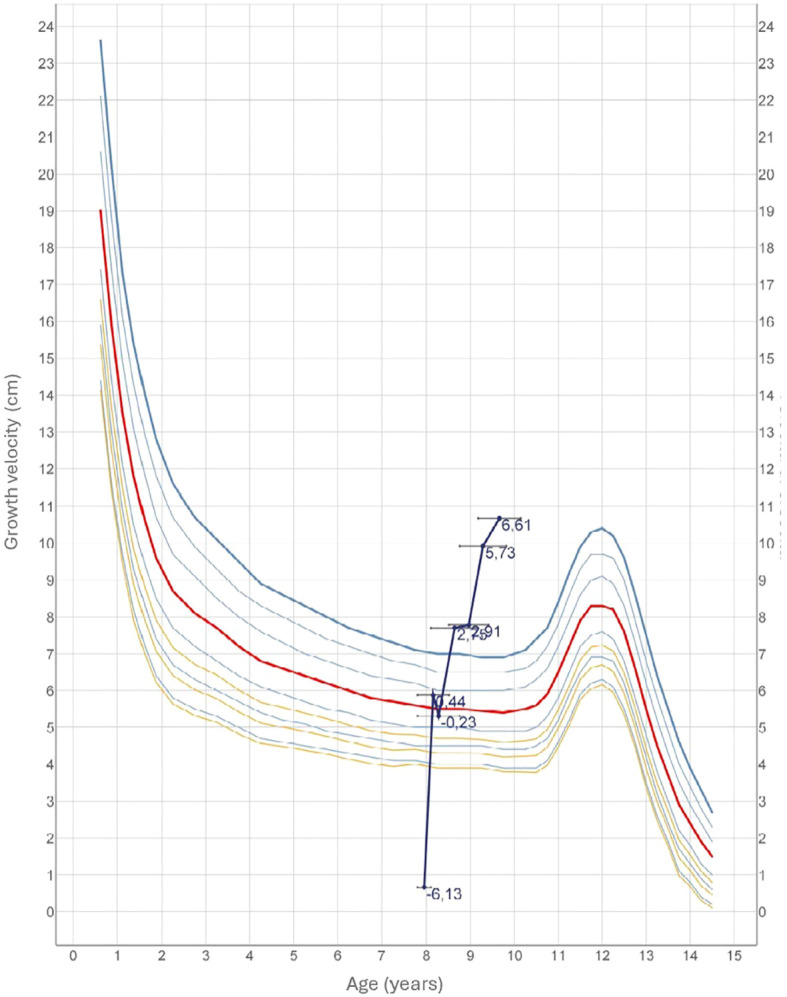
Growth Velocity SDS using Tanner 1966 growth charts.

**Figure 3:**
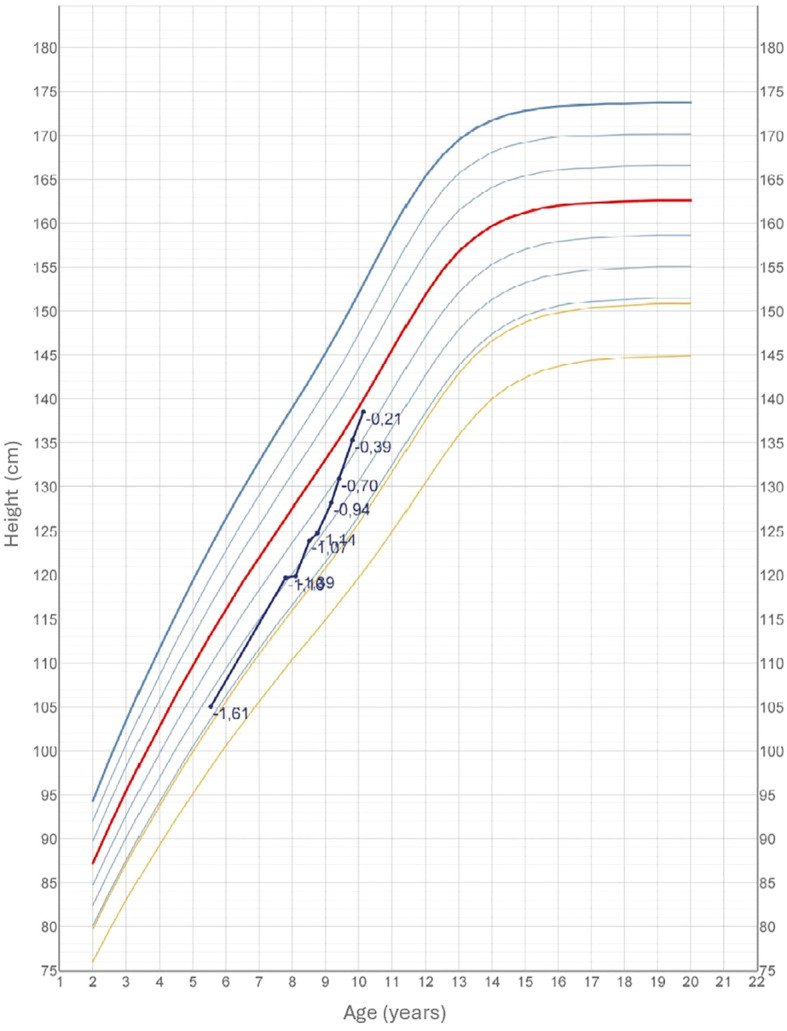
Height SDS using Cacciari 2006 growth charts for Italian girls.

**Table 3:** Timeline of clinical and laboratory features at GHH diagnosis and follow-up.

Clinical and laboratory features of GHH diagnosis and follow-up						Normal values
Age	8.6	9.1	9.4	9.8	10.1	
Height (cm)	124.8 (-1.14)	128.2 (-0.95 SDS)	130.9 (-0.70 SDS)	135.3 (-0.39 SDS)	138.5 (-0.21 SDS)	
Weight (kg)	25.8 (-0.37)	31 (+0.43 SDS)	33.2 (+0.65 SDS)	36.1 (+0.82 SDS)	35.6 (+0.52 SDS)	
BMI (kg/m^2^)	16.6 (+0.30 SDS)	18.9 (+1.15 SDS)	19.4 (+1.27 SDS)	19.7 (+1.28 SDS)	18.6 (+0.79 SDS)	
Tanner stage – Breast (B)	1	2	3	3	4	
Tanner stage – Pubic hair (PH)	1	1	1	2	2	
Growth velocity (cm/year)	7.48 (+2.48 SDS)	7.7 (+2.86 SDS)	7.8 (+3.04 SDS)	9.9 (+5.65 SDS)	10.7 (+3.59 SDS)	
Systolic blood pressure (mmHg)	114 (+1.58 SDS)	115 (+1.67 SDS)	110 (-1.08 SDS)	110 (+1 SDS)	111 (+1 SDS)	
Diastolic blood pressure (mmHg)	69 (+1.08 SDS)	66 (+ 0.67 SDS)	64 (+0.42 SDS)	63 (+0.25 SDS)	73 (+1.17 SDS)	
Bone age (years)	8.6	---	---	---	11.1	
Thyroid US	Nodule 0.4 cm in lower right lobe	Nodule 0.4 cm in lower right lobe	Nodule 0.5 cm in lower right lobe	Nodule 0.5 cm in lower right lobe	---	
Ecocardiography	Normal	---	---	Normal	---	
IGF-1 (ng/mL)	464.8 (+2.42 SDS)	498.2 (+2.25 SDS)	399.3 (+1.61 SDS)	529.3 (+2.44 SDS)	430.1 (+1.5 SDS)	8 years: 39-396 9 years: 49-451 10 years: 62-504
TSH (µUI/ml)	1.388	3.626	---	2.339	2.809	0.20-6.1
FT4 (pg/mL)	16.8	10.2	---	9.6	8.8	6.9-16.9
PRL (ng/ml)	9.1	----	---	---	----	<25
ACTH (pg/ml)	3.3	3.4	3.3	2.2	25.3	<60
LH (UI/L)	0.3	---	---	1.8	1.3	Prepubertal: <0.3
FSH (UI/L)	4.5	---	---	7.8	6.7	0.3-10
17-beta-estradiol (pg/mL)	0			20.5	34.5	0-16
Glucose (mg/dl)	64	73	72	72	89	60-100
HbA1c (%)	---	---	5.6	---	---	4.3-6.1
Total cholesterol (mg/dl)	153	153	146	160	114	60-200
LDL (mg/dl)	91	84	80	89	59	10-129
Triglycerides (mg/dl)	53	43	51	56	53	20-175

Height SDS refer to Cacciari 2006 growth charts for Italian girls. BMI and weight SDS refer to WHO 2006 growth charts for girls >5 years. Growth velocity SDS refer to Tanner 1966 growth charts and are adjusted for bone age when available. Bone age was calculated using Tanner-Whitehouse 2 method. Blood pressure SDS refer to (6).

At the most recent follow-up after 18 months since diagnosis of GHH, the patient still showed accelerated GV (+3.59 SDS adjusted for bone age), accompanied by progression of pubertal development while IGF-1 levels were within the normal range for sex and age (+1.5 SDS). Bone age was advanced by one year (10.1 years chronological age vs 11.1 years bone age analyzed with TW2) and echocardiography showed preserved cardiac function, and absence of cardiac myxomas. Given the negative MRI findings, age-appropriate IGF-1 levels, and normal metabolic parameters without evidence of cardiovascular comorbidities or neoplasia as assessed during follow-up visits, we decided to continue with clinical and laboratory monitoring.

## 3 Discussion

The patient we described in our report presented with the typical signs of CS (rapid weight gain associated with changing in facial physiognomy, acne, hypertrichosis, asthenia, GV decrease and hypertension), and some rare manifestations in pediatrics such as hypokalemic alkalosis and kidney stones.

CS is a rare disease, with an incidence of 0.7–2.4 cases per million people per year. Only about 10% of all cases occur in children (10, 11), and among these, ACTH-independent CS accounts for approximately 15% (10). ACTH-independent forms include adrenocortical tumors, which represent the most common causes of adrenal CS in pediatric patients, and primary adrenal hyperplasia, which includes PPNAD, primary bilateral adrenal macronodular hyperplasia (PBAMH) and McCune Albright Syndrome (MAS) (11). These forms of adrenal hyperplasia are frequently associated with genetic syndromes, such as CNC and MAS (12). Therefore, in any case of ACTH-independent CS in children, it is essential to consider the possible presence of an underlying genetic condition. In our case, MAS appeared unlikely due to the absence of the clinical characteristics associated with the syndrome (13, 14). Furthermore MAS-associated hypercortisolism has been mainly described in neonatal period and is uncommon after the first year of life (12, 13, 15). These evidences as well as the patient’s family history were fundamental to direct our diagnostic suspicion toward CNC (11).

PPNAD, together with cardiac and cutaneous myxomas, constitutes one of the most frequent clinical manifestations of CNC in pediatric patients and is the most common endocrine tumor described in the total CNC population since it occurs in about a quarter of patients (2, 4). It is characterized by multiple pigmented nodules surrounded by an atrophic cortex that autonomously secrete cortisol and can lead children and adolescents with PPNAD to have periodic or cyclical CS (10). Clinical presentation of CS caused by PPNAD is often characterized by signs of hyperandrogenism and osteoporosis together with other symptoms of CNC (11). PPNAD is often difficult to identify using radiological studies, as the adrenal glands typically appear normal or small in size (16). This makes CT and MRI more effective than ultrasound in detecting the lesion (11). In our case, MRI was preferred because it avoids ionizing radiation exposure, allows multiplanar imaging, and provides superior soft-tissue contrast resolution (11).

The main diagnostic challenge in this case was the absence of hallmark cutaneous manifestations of CNC, such as lentigines or myxomas. Careful family history evaluation was pivotal in generating clinical suspicion for CNC. This, correlated with ACTH-independent CS without visible masses on first level imaging, prompted targeted second level adrenal imaging that successfully identified bilateral PPNAD supporting clinical suspicion of CNC.

In pediatric patients, CNC presents with some distinct early-onset features. Lentigines and other pigmented skin lesions may be visible since birth, but classically increase in number during puberty, often constituting the first clinical sign of the disorder (1, 2, 4). Cutaneous myxomas also appear in childhood, particularly affecting the eyelids, ears, and nipples (4). LCCSCTs are often identified in the first decade of life in males and may be associated with prepubertal gynecomastia due to aromatase hyperactivity. Thyroid nodules, typically benign, are frequently detected in adolescence. While cardiac myxomas can present at any age, they are often diagnosed in childhood in CNC patients and require early echocardiographic surveillance due to the associated risk of embolic events or sudden death. These age-specific patterns emphasize the importance of targeted early screening and vigilant follow-up in pediatric patients with CNC (1, 2, 4).

A distinctive feature in our patient at the time of diagnosis was the presence of kidney stones, which has been reported in only one previous case of CNC (17). Nevertheless, nephrolithiasis is a well-recognized complication of CS in adults, with studies indicating that approximately 50% of adult patients with Cushing’s disease develop kidney stones (18, 19).

The development of renal calculi is probably related to an increased bone resorption that occurs in patients with high levels of cortisol, including in children with CS, which likely contributes to their hypercalciuria (20, 21). Moreover, hypercortisolemia can lead to hyperuricosuria and hypercystinuria, further increasing the risk of kidney stone formation (22).

Asymptomatic GHH and IGF-1 secretion in CNC presents in 30 to 75% of affected patients, while only 15 to 18.9% develop acromegaly (2, 5); gigantism caused by GHH prior to puberty is rare (2). In 2024 Tatsi et al. studied the spectrum of GHH in a cohort of 140 patients with CNC; GHH was present in 50 patients (35,7%), with a median age at diagnosis of 25.3 years and clinical acromegaly was described in 28 patients (20%). The youngest patient was 9.5 years old, and 13 out of 50 patients (26%) were diagnosed with GHH before the age of 18. These findings lead the authors to the recommendation to initiate GHH screening with IGF-1 measurement at the age of 9, or at the onset of the earliest pubertal signs (5).

To the best of our knowledge, this appears to be the earliest case of GHH onset in childhood reported in literature. The only other documented case of childhood-onset GHH was included in the study by Tatsi et al. and involved a patient aged 9.5 years. It is unclear whether the aforementioned patient exhibited any signs of pubertal development.

The presence of acromegaly with lack of evident clinical signs was also described in a study by Espiard et al. in 2020 that analyzed a cohort of 70 patients with CNC. Prevalence of acromegaly in this cohort of patients was 18.6%, all of the patients affected presented with mild or absent clinical signs of acromegaly (23). Another interesting feature of our patient is the hypersecretion of IGF-1 and the abnormal OGTT in absence of a pituitary adenoma on brain MRI. In 2000 Pack et al. described 8 patients with CNC and acromegaly and 4 of these patients presented abnormalities of GH secretion before radiological evidence of a pituitary tumor (24). This finding may be attributable to a characteristic frequently described in patients undergoing surgery for pituitary adenoma secondary to CNC, which is the histological detection of multiple small hyperplastic areas identified in the extratumoral pituitary parenchyma, composed of GH-secreting cells (1, 24–27). This supports the hypothesis that cellular hyperplasia might be the precursor of the actual adenoma (25, 27) explaining the protracted period of onset of clinically evident acromegaly (2).

Regarding treatment of children with CNC and GHH without pituitary abnormalities there are few evidences in literature, and almost all of these mainly focus on adult patients leaving the choice of the treatment to the clinician’s judgement. In the aforementioned study by Tatsi et al. the management of patients with CNC and GHH was mainly individualized considering individual characteristics such as age at diagnosis, degree of GHH, MRI findings and the presence of comorbidities such as cardiac myxomas. Of the 28 patients with clinically significant GHH, 13 received transsphenoidal surgery, 11 received medical treatment and 4 had a clinical monitoring (5). In 2006 Boikos et al. analyzed a smaller cohort of patients with CNC and acromegaly aged from 16 to 70 years stating that their practice was to treat all the patients with clinical acromegaly, but negative pituitary imaging with somatostatin analogues, with the aim of normalizing IGF-1 levels (25). This therapeutic approach could be reinforced by evidence of a correlation between GHH and the development of cardiac myxomas in patients with CNC, as highlighted in a 2016 study by Bandettini et al. (28).

In our case, the absence of cardiac involvement or neoplasia, together with the normalization of IGF-1 levels and persistently normal MRI findings, supported the decision to defer medical treatment. Furthermore, normalization of IGF-1 across two different measurements led us to hypothesize that GHH may represent a transient or at least inconstant phenomenon, as described in other conditions such as Neurofibromatosis Type1(NF1) with optic pathway gliomas (OPG) (29). In this condition, the mechanism underlying GHH remains unclear. Patients typically exhibit a normal pituitary gland, leading to the hypothesis of suppressed somatostatin tone in the presence of OPG (30). GHH in NF1 has been described as a transient phenomenon which resolves regardless of treatment; benefits of the treatment on outcomes or prevention of tumor progression are still unclear (29). Given the current lack of specific recommendations for the management of pediatric CNC patients with GHH and normal pituitary imaging, close and dedicated follow-up remains essential to promptly identify disease progression and guide therapeutic decisions.

The overall clinical course is summarized in **Figure 4**.

**Figure 4:**
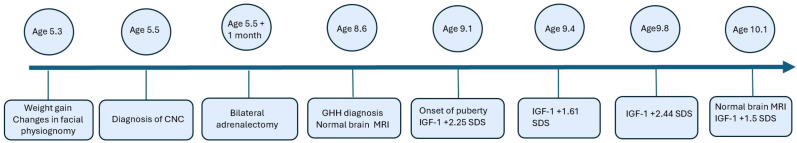
Timeline.

## 4 Study limitations

This case report has several limitations. The 18-month follow-up, while adequate to document early GHH and subsequent IGF-1 normalization, remains relatively short to assess the long-term natural history of GHH. Finally, generalizability is intrinsically limited by the single-case design.

## 5 Conclusion

When CS is diagnosed in children, especially without adrenal mass, genetic diseases such as CNC should be considered. Clinicians should always pay careful attention to family history through an accurate medical history assessment, in order to identify any elements that may indicate the need for genetic testing.

Although current guidelines recommend initiating hormonal screening for pituitary adenoma in CNC starting in adolescence (2, 31), our case highlights the importance of a careful monitoring of GV. The first sign of acceleration in GV should prompt the initiation of screening for GHH in pediatric patients with CNC at any age.

## Data Availability

The data supporting the findings of this study are not publicly available due to patient privacy and ethical restrictions but are available from the corresponding author upon reasonable request.
